# Isolation of circulating tumor cells from pancreatic cancer by automated filtration

**DOI:** 10.18632/oncotarget.21026

**Published:** 2017-09-16

**Authors:** Nora Brychta, Michael Drosch, Christiane Driemel, Johannes C. Fischer, Rui P. Neves, Irene Esposito, Wolfram Knoefel, Birte Möhlendick, Claudia Hille, Antje Stresemann, Thomas Krahn, Matthias U. Kassack, Nikolas H. Stoecklein, Oliver von Ahsen

**Affiliations:** ^1^ Bayer AG, Biomarker Research, 13353 Berlin, Germany; ^2^ Department of General, Visceral and Pediatric Surgery, Medical Faculty, University Hospital of the Heinrich-Heine-University Duesseldorf, 40225 Duesseldorf, Germany; ^3^ Institute of Pathology, Heinrich-Heine-University of Duesseldorf, 40225 Duesseldorf, Germany; ^4^ Institute of Pharmaceutical & Medicinal Chemistry, University of Duesseldorf, 40225 Duesseldorf, Germany; ^5^ Current/Present address: University Medical Center Hamburg-Eppendorf, Department of Tumor Biology, 20246 Hamburg, Germany; ^6^ Current/Present address: JPT Peptide Technologies GmbH, 12489 Berlin, Germany

**Keywords:** pancreatic cancer, circulating tumor cells, *KRAS*, diagnostic leukapheresis, EMT

## Abstract

It is now widely recognized that the isolation of circulating tumor cells based on cell surface markers might be hindered by variability in their protein expression. Especially in pancreatic cancer, isolation based only on EpCAM expression has produced very diverse results. Methods that are independent of surface markers and therefore independent of phenotypical changes in the circulating cells might increase CTC recovery also in pancreatic cancer. We compared an EpCAM-dependent (IsoFlux) and a size-dependent (automated Siemens Healthineers filtration device) isolation method for the enrichment of pancreatic cancer CTCs. The recovery rate of the filtration based approach is dramatically superior to the EpCAM-dependent approach especially for cells with low EpCAM-expression (filtration: 52%, EpCAM-dependent: 1%). As storage and shipment of clinical samples is important for centralized analyses, we also evaluated the use of frozen diagnostic leukapheresis (DLA) as source for isolating CTCs and subsequent genetic analysis such as *KRAS* mutation detection analysis. Using frozen DLA samples of pancreatic cancer patients we detected CTCs in 42% of the samples by automated filtration.

## INTRODUCTION

Pancreatic cancer is among the most lethal cancer diseases worldwide and the cases of newly diagnosed patients will raise further in the following years due to the demographic changes in the developed countries [1]. The 5-year survival rate of patients diagnosed with pancreatic cancer is still below 10% [2]. Major reason for this is the lack of effective screening for early detection and the usually late diagnosis due to nonspecific symptoms late in the progress of the disease. Nowadays, resection of the tumor is the only curative treatment. As metastasis occurs after initial tumor progression [3], early detection is of utmost importance for successful treatment. Pancreatic cancer seems to disseminate tumor cells relatively early as it has been shown that patients undergoing pancreatectomy for tumors smaller than 2 cm have less than 18% 5 years survival [4]. Even some patients after pancreatectomy for chronic pancreatitis develop disseminated pancreatic ductal adenocarcinoma (PDAC) although no tumors were found in the primary resectate [5]. However, until today no early screening tests are in routine clinical use [6]. CA19.9 is the only biomarker used to support diagnosis and response monitoring but is not sensitive enough for early detection [7].

One alternative diagnostic tool might be blood-based biomarkers like circulating tumor cells (CTCs). The relevance of CTCs in pancreatic cancer has recently been reviewed [8, 9]. CTCs originate from the tumor, are shed from tissue into the blood stream and may be representative of the systemic disease [10]. On the other hand, CTCs are rare cells occurring in very low concentration in the peripheral blood which makes their detection challenging [11]. Several methods for isolation of CTCs have been used to isolate these cells utilizing different characteristics of the tumorous cells like surface marker or size [12]. One widely used surface marker is the epithelial cell adhesion molecule (EpCAM). EpCAM is expressed on cells derived from epithelial tumors including CTCs but not on regular blood cells such as leukocytes [13]. Since the EpCAM-based isolation of CTCs (CellSearch) is FDA-cleared for metastatic breast, prostate and colorectal cancer, it is the gold standard for CTC research and the number of CTCs was already described as prognostic for survival in these indications [14–16].

Although most pancreatic tumors are EpCAM-positive (96%) [17], the expression levels of EpCAM are heterogenous with only half of the tumors showing strong expression [18, 19]. This may explain why both the number of EpCAM-captured CTCs and also the number of CTC-positive patient cases is low [13, 20, 21]. The original publication on the CellSearch-Device describes the CTC-numbers in PDAC as among the lowest of all indications even in samples from metastatic patients compared to breast, colorectal or prostate cancer [13]. Although most studies showed comparably low numbers of CTCs in pancreatic cancer, the reported detection rates range from 5-100% depending on the volume of blood, isolation method and staining technique [20, 22–24]. This strong variability in the results hints to the strong need of better definition of CTC properties and careful validation of the technologies used.

A major obstacle for EpCAM-based CTC isolation is the epithelial-mesenchymal-transition (EMT) often observed with CTCs [25–27]. For pancreatic cancer this has already been described *in vivo* showing the loss of epithelial markers at an early stage of development [28]. Therefore, an antigen-dependent approach for CTC isolation is especially difficult in pancreatic cancer [23, 29]. In addition to EMT other mechanisms of EpCAM downregulation such as internalization, proteolysis and promotor methylation have been described that may reduce the success rate of CTC isolation, as reviewed by Gires and Stoecklein [30].

Consequently, antigen-independent capturing strategies of CTCs emerged to overcome the challenge of detecting all phenotypic variants of CTCs. One possible alternative to the immune-affinity purification might be the filtration of CTCs. A pilot study by Khoja *et al.* already showed very promising results by using a filtration method (ISET) in pancreatic cancer [31]. In order to increase throughput and standardize handling, automation is the ultimate goal for clinical devices. Therefore we tested an automated filtration-platform produced by Siemens Healthineers [32].

Since the enumeration of CTCs solely based on EpCAM expression (or that of other epithelial markers like cytokeratins) may not be sufficient for unequivocal identification of cancer cells due to EMT, downstream analysis becomes important for defining truly tumor-derived cells. In pancreatic cancer, *KRAS* mutations are commonly used to detect malignant cells, because of their high prevalence of 57% [33]. This has been successfully used for CTCs isolated by other techniques [22] but one group also reported non-matching *KRAS* status between tumor and CTCs in some cases [34].

In this work, we compare two different isolation techniques with automated devices (EpCAM-dependent immune-affinity purification versus filtration) for capturing of CTCs in whole blood. A proof of concept study with frozen diagnostic leukapheresis samples (DLA) was performed to determine the possibility of improved CTC detection in stored frozen patient samples followed by *KRAS*-mutational analysis.

## RESULTS

**Figure 1:**
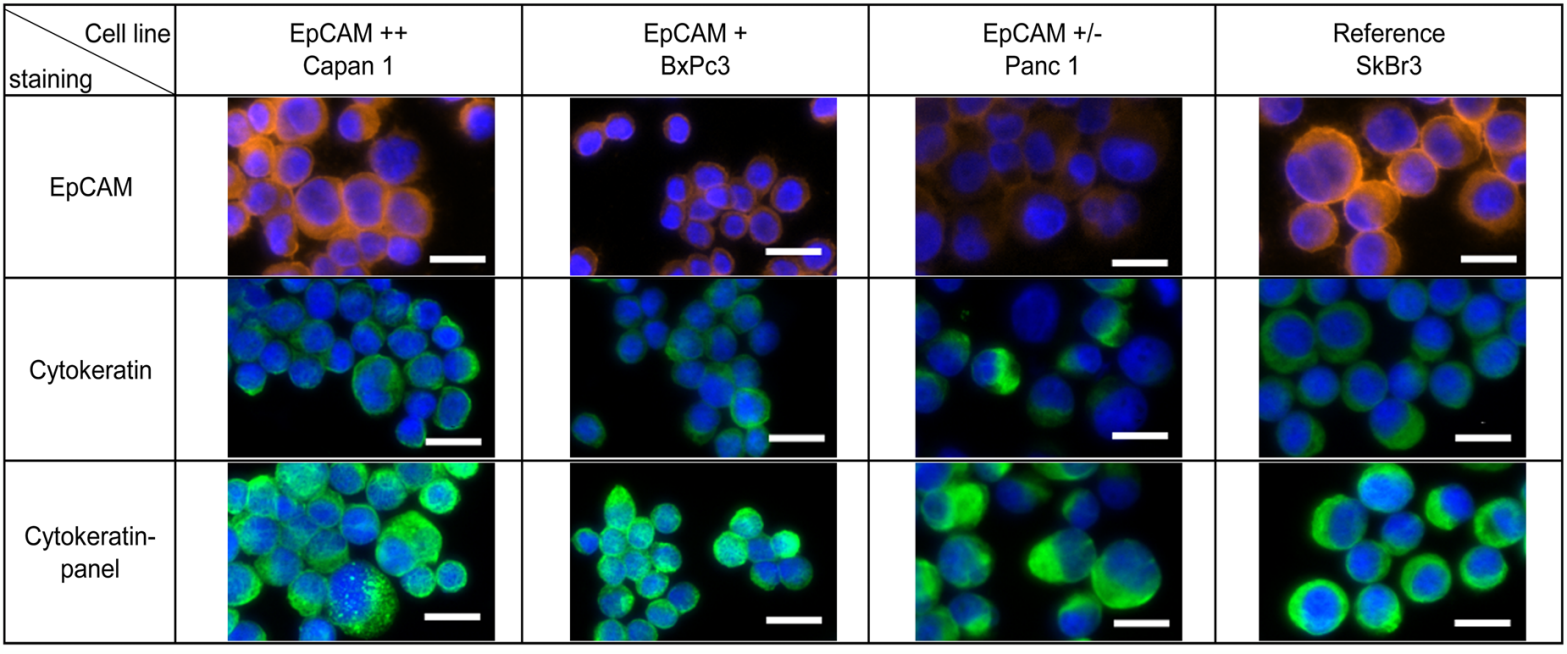
Cytokeratin expression in pancreatic cancer Pancreatic cancer cell lines Capan1, BxPc3, Panc1 and breast cancer cell line SkBr3 were characterized by immunofluorescence staining with anti-EpCAM (Alexa Fluor 555, orange) and anti-Cytokeratin (Alexa Fluor 488, green). Nucleus was stained by Hoechst 33342 (blue). Scale bars represent 20μm.

Here we compared two methods for isolation of circulating tumor cells from pancreatic cancer. Cultured cells spiked in whole blood were used to test the performance of the methods. As EpCAM expression is highly variable within CTC-populations we selected three different pancreatic cancer cell lines that express different levels of EpCAM for the required spike-in experiments (Figure 1): Capan1 as high, BxPc3 as medium and Panc1 as low EpCAM expressing cell lines. The breast cancer cell line SkBr3 was additionally used as reference cell line as it was frequently used for the evaluation of analytical devices [32, 35]. Detection of CTCs is routinely performed by cytokeratin staining as marker for the epithelial origin of the cells. Therefore, we also included cytokeratin stainings in Figure 1. In addition to differences in EpCAM expression, cytokeratin levels also vary between cell lines. Pancreatic cancer cells express predominantly cytokeratins 7, 8, 13, 18 and 19 (Supplementary Table 1). Most pan-cytokeratin antibodies like AE1/AE3 miss one or more of these cytokeratins. Therefore we developed an antibody mix that covers all the cytokeratins expressed in pancreatic cancer (Supplementary Table 2). By using this panel of cytokeratin antibodies, we enhanced the signal for all 4 cell lines tested to a similar fluorescence intensity level independent of the cytokeratin isoforms expressed (Figure 1).

**Figure 2:**
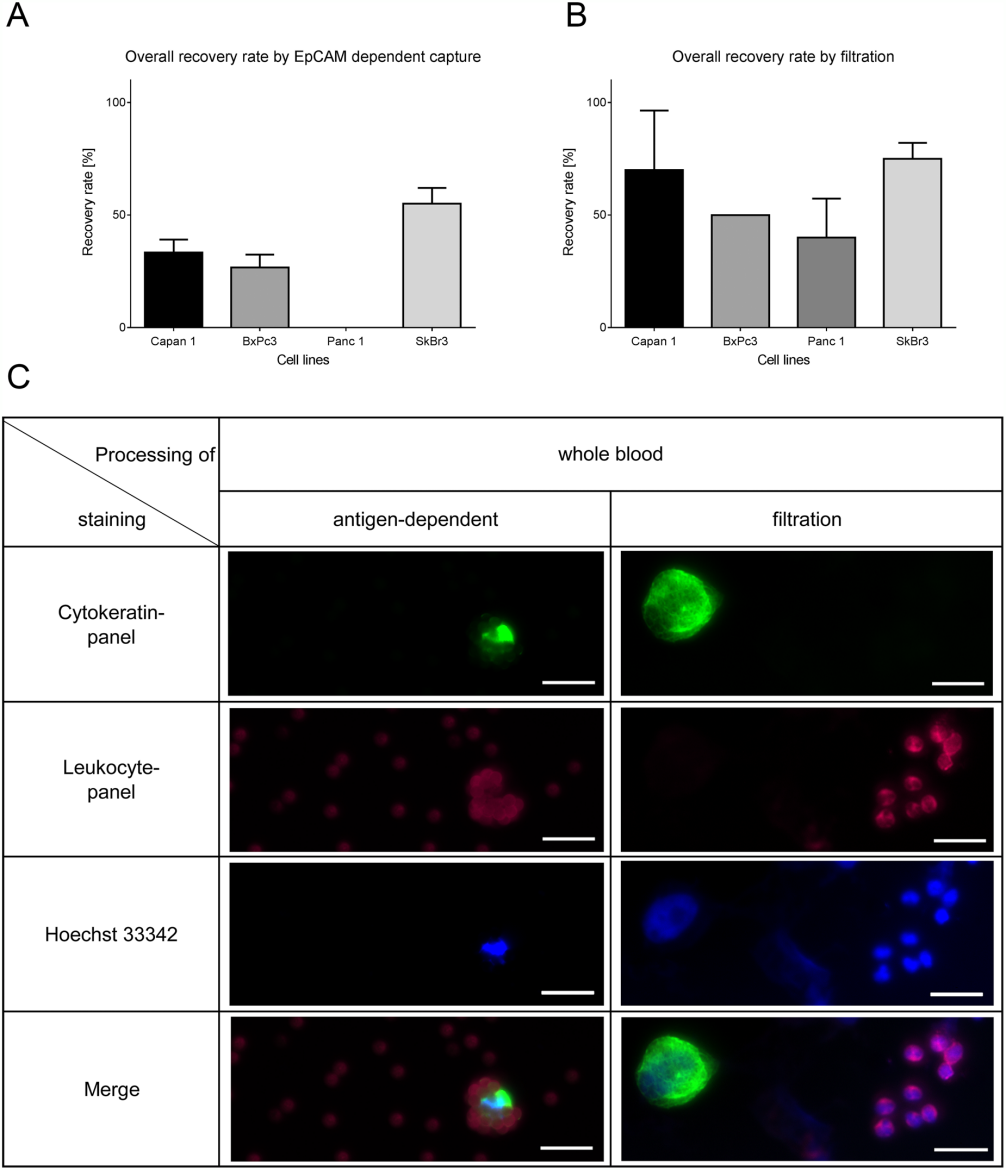
Recovery rates by filtration and EpCAM-dependent isolation method of spiked cells in whole blood (10 cells of each cell line in triplicates) **(A)** Recovery rates by EpCAM-dependent magnetic bead isolation is lower in pancreatic cancer cells compared to SkBr3. **(B)** The filtration method shows high recovery rates independent of the EpCAM-expression. **(C)** Representative pictures of Capan1. The filtration also helps to identify morphology whereas the beads overshadow the signal of the immunofluorescence staining. Cells were stained by anti-Cytokeratin (green), anti-leukocyte panel (magenta) and nucleus (blue). Scale bars represent 20μm.

Next we determined the performance of two different CTC isolation methods by spiking cells into whole blood. We used an EpCAM-dependent immune-affinity approach and a filtration method to examine if the variable EpCAM expression in pancreatic cancer cells has an impact on the recovery rate. One, 3, 10 and 30 cells each were spiked in whole blood and subjected to the respective isolation procedures. The average recovery rate in the IsoFlux device is highly dependent on EpCAM expression (Figure 2A shows the result for 10 spiked cells and Supplementary Figure 1 for all numbers of spiked cells) compared to the size-dependent method which recovered in average more than 50% of the spiked cells independent of the EpCAM expression (Figure 2B shows the results for 10 spiked cells and Supplementary Figure 1 for all cell numbers tested). Therefore filtration gives a much more robust result throughout the variety EpCAM-expression in cell lines. Before subjecting the sample to the IsoFlux device, whole blood has to be separated by a Ficoll density gradient. This is part of the standard procedure recommended by Fluxion, but might result in a loss of CTCs which has been previously reported [13, 36]. We also tested the potential loss of CTCs in the Ficoll gradient and observed losses compared to spiking after the Ficoll gradient (data not shown). As the used beads are rather big, even the staining might be obscured by the beads (Figure 2C and Supplementary Figure 3) and hinder the detection. Figure 2C displays representative pictures of Capan1 cells isolated by the two different methods (see also Supplementary Figures 2 and 3 for all cell lines). Cells on the filters show better visual quality compared to the cells covered with magnetic beads which disturb the imaging. The detection rate of the breast cancer cell line SkBr3 was higher in both methods (mean IsoFlux: 49%, mean filtration: 80%) owing to the very high EpCAM expression and the large cell size compared to the pancreatic cancer cell lines. We conclude that filtration is superior to surface antigen-dependent isolation of CTCs.

**Figure 3:**
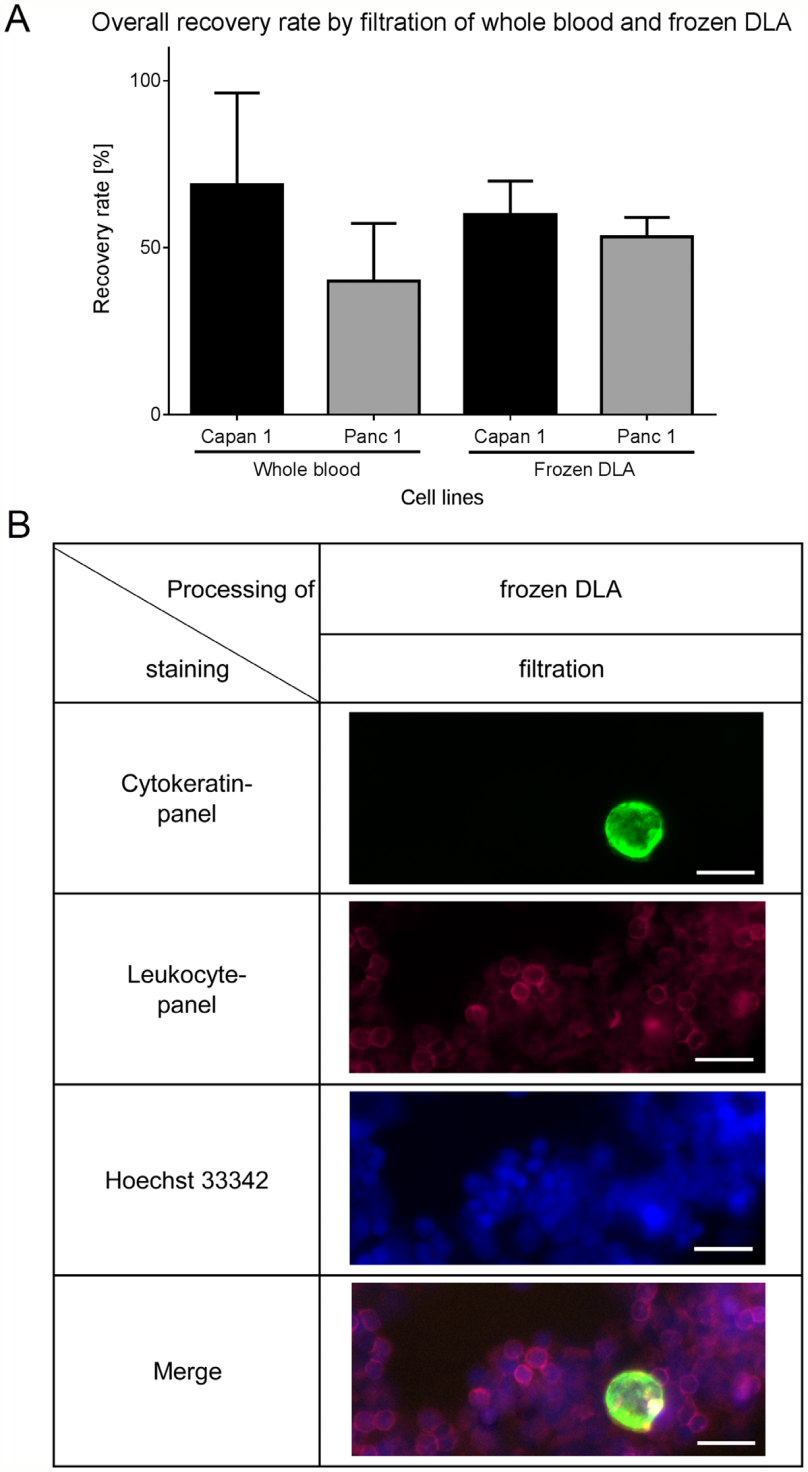
Recovery rates by filtration of spiked cells in frozen DLA **(A)** Recovery rates of the thawed DLA are similar to the results of filtration of whole blood (n=12). **(B)** Representative pictures of Capan1 isolated from frozen DLA by filtration. Cells were stained by anti-Cytokeratin (green), anti-leukocyte panel (magenta) and nucleus (blue). Scale bars represent 20μm.

As whole blood samples cannot be stored and shipped easily and also to increase the amount of input material, we examined another clinical sample type, frozen apheresate, originating from diagnostic leukapheresis (DLA). DLA is a blood preparation highly enriched in mononuclear cells. It has been shown that CTCs co-enrich in the mononuclear fraction [11]. In the present study 2mL of DLA product correspond to 50mL whole blood sample in terms of white blood cell counts. The DLA-product can be frozen and is therefore ideal for potential use in larger clinical studies. To test the performance of filtration after freezing and thawing of the DLA product we tested the recovery rate of Capan1 and Panc1 cells spiked in healthy donor DLA product. The detection rate of the isolated cells was similar to those isolated from whole blood (Figure 3A for 10 spiked cells and Supplementary Figure 4 for different cell numbers). When cultured cells were spiked in freshly prepared buffy coat, frozen, stored for several weeks and thawed using our established protocol the cell recovery was similar (Supplementary Figure 5). Representative pictures also show that cells stay intact during thawing (Figure 3B) as the morphology is similar to the cells isolated from whole blood (Figure 2C). Therefore the use of frozen DLA product as a source for CTC isolation is feasible.

After validation of input material and the pre-analytical procedures we next used the filtration method to analyze DLA samples of pancreatic cancer patients. We detected CTCs in 42% (8/19) of the patient samples. We did not find a higher prevalence of CTC positivity in metastatic cases: 44% in M1 (4/9) and 40% in M0 (4/10) (Table 1). However, higher numbers of CTCs were found in patients diagnosed with distant metastasis.

**Table 1:** Number of detected CTCs isolated from frozen DLA of pancreatic cancer patients in sample set A (2 vials, 2 x 10^8^ cells/mL). Overall detection rate of CTCs was 42% (44% M1, 40% M0). No significant correlations with any clinical parameter were found

Sample ID	Tumor type	Stage	Grade	CTCs	Histology	Age	Sex
5931	Pancreatic	2a	2	0	moderately differenciated ductal adenocarcinoma	57	m
5804	pancreatic	2b	2	2	moderately differenciated adenocarcinoma	69	m
5549	pancreatic	2b	2	2	moderately differenciated ductal adenocarcinoma	52	f
5714	pancreatic	2b	3	0	poorly differenciated adenocarcinoma	86	m
5751	pancreatic	2b	2	0	moderately differenciated ductal adenocarcinoma	54	m
5902	pancreatic	2b	2	0	moderately differenciated ductal adenocarcinoma	73	m
6033	pancreatic	2b	3	1	poorly differenciated ductal adenocarcinoma	63	m
6104	pancreatic	2b	2	0	moderately differenciated ductal adenocarcinoma	55	f
5689	pancreatic	2b	3	2	moderately differenciated ductal adenocarcinoma	55	m
6017	pancreatic	3	2	0	moderately differenciated ductal adenocarcinoma	70	f
5447	pancreatic	4	2	0	moderately differenciated mucinous ductal adenocarcinoma	72	m
5580	pancreatic	4	2	0	moderately differenciated ductal adenocarcinoma	51	f
5792	pancreatic	4	3	1	poorly differenciated ductal adenocarcinoma	72	f
6012	pancreatic	4	3	0	poorly differenciated ductal adenocarcinoma	66	f
6098	pancreatic	4	2	0	moderately differenciated ductal adenocarcinoma	54	f
6016	pancreatic	4	3	0	poorly differenciated adenocarcinoma	58	f
5539	pancreatic	4	3	7	poorly differenciated adenocarcinoma	85	m
5803	pancreatic	4	3	2	poorly differenciated ductal adenocarcinoma	72	f
5904	pancreatic	4	2	7	moderately differenciated ductal adenocarcinoma	73	f

**Figure 4:**
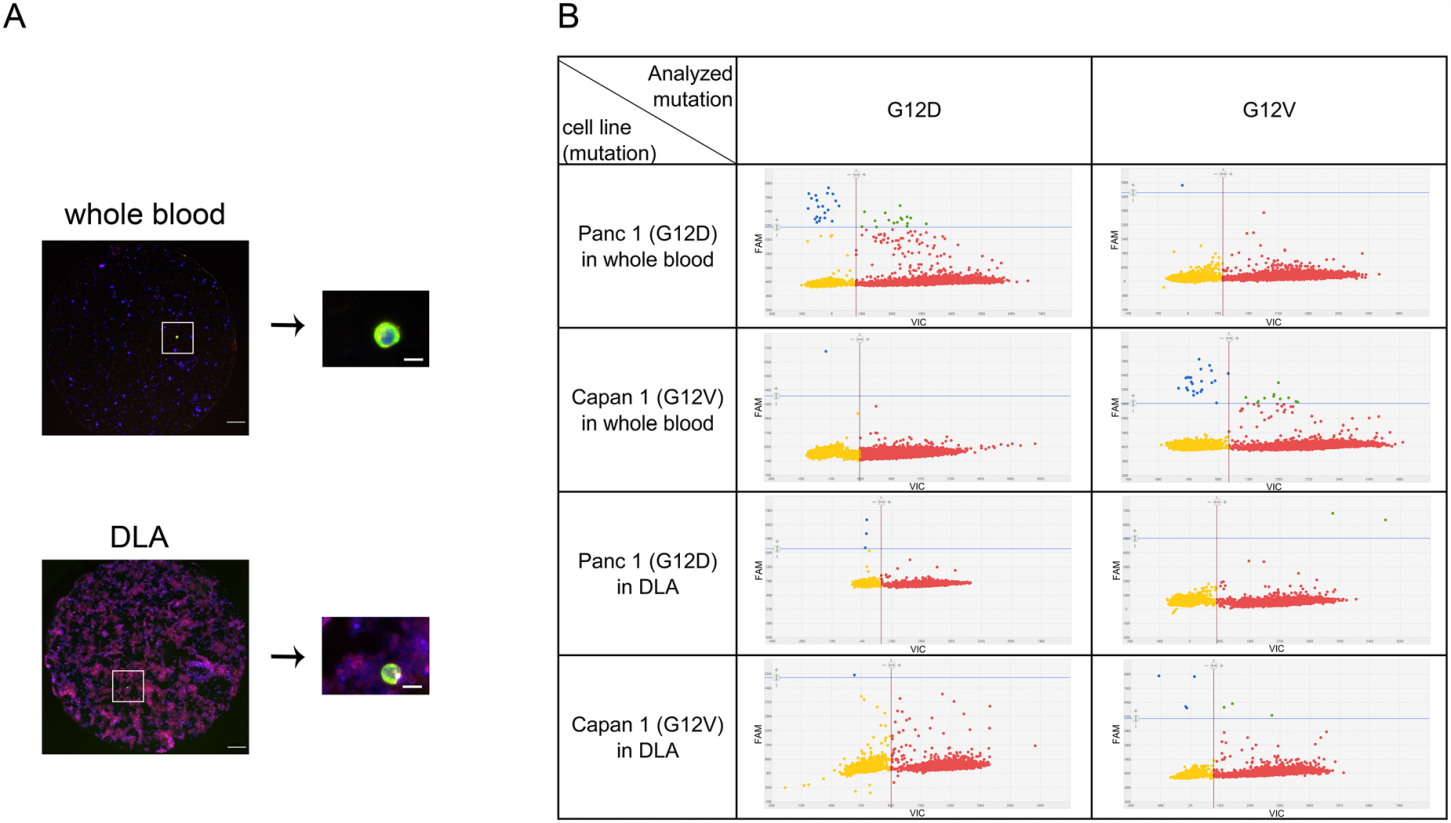
Mutation detection of CTCs in whole blood and frozen DLA Red dots represent wild-type, blue dots the mutation, microwells with both signals are colored in green and microwells without signal yellow. **(A)** Detected cells were punched out from filter and relocated on the punch before WGA. **(B)** Amplified DNA was analyzed by digital PCR for *KRAS* G12D and *KRAS* G12V. Note higher background of leukocytes in DLA. Scale bars represent 200μm (filter) and 20μm (single cell).

In order to confirm the identity of the isolated cells as cancer derived CTCs we developed a method for mutational analysis of isolated CTCs picked from the filter (Figure 4). Pancreatic cancer cells were spiked into whole blood and DLA of healthy donors, filtered and stained. After enumeration of the cells by fluorescence microscopy, we punched out single cells and subjected them to whole genome amplification (WGA) followed by digital PCR analysis for *KRAS* G12D and G12V. *KRAS* mutations were detected in both punches of whole blood and punches of DLA spiked with Capan1 (G12V homozygous) or Panc1 (G12D heterozygous) (Figure 4). It is evident that the increased number of white blood cells in the DLA-product causes more background on the filter stained for cytokeratin (Figure 4A) as well as in the dPCR analysis (Figure 4B). The large input of wild-type alleles from the white blood cells dominates the result. Nevertheless, mutant cells can be detected. The analysis of punches derived from healthy blood or DLA without spiked cancer cells showed no evidence for KRAS mutations proving the specificity of the assay (data not shown).

**Figure 5:**
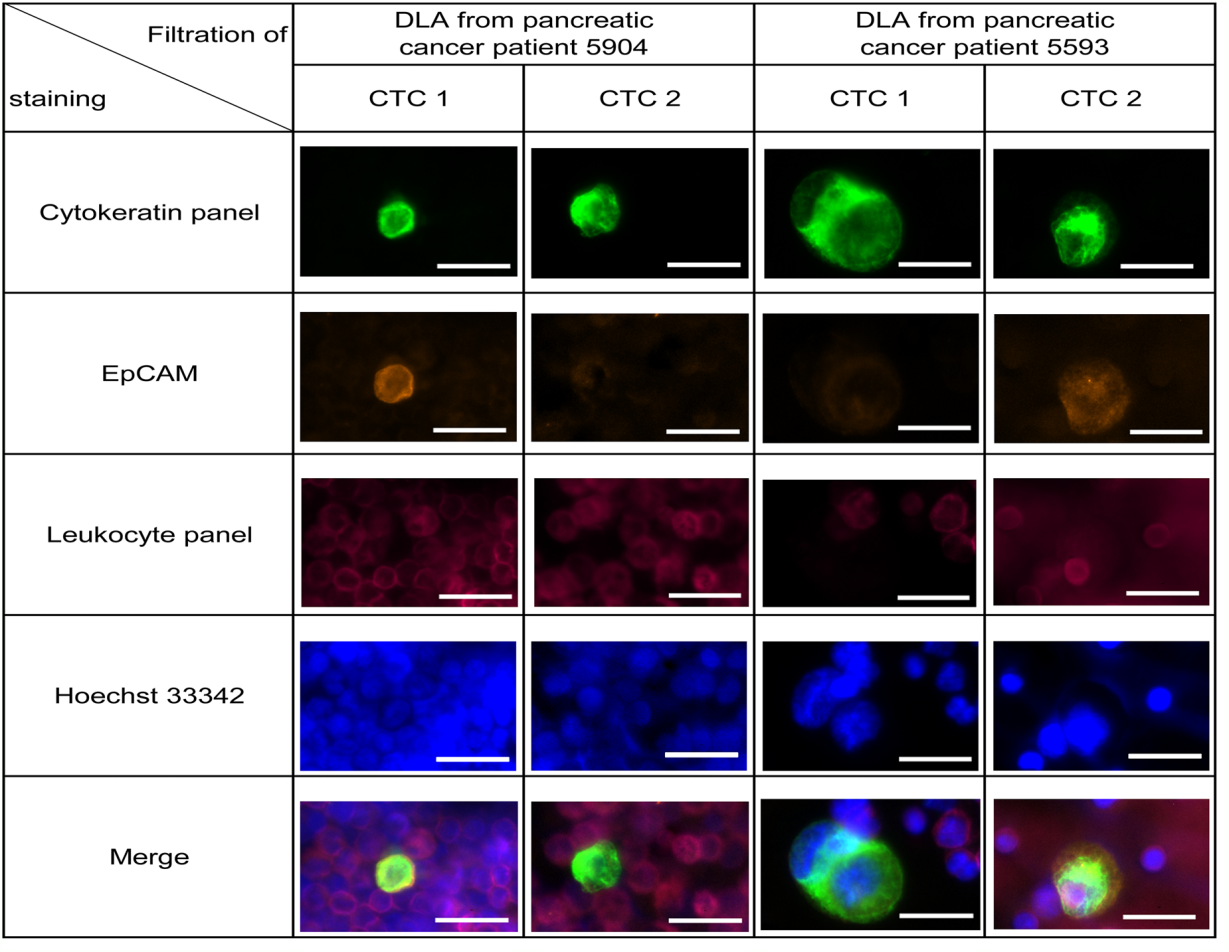
Detection of CTCs in frozen DLA samples from 19 pancreatic cancer patients Representative pictures of isolated cell from patient 5904 and 5593. Within one patient both EpCAM high and EpCAM low CTCs were detected (see also Supplementary Figure 6). Cells were stained by anti-Cytokeratin (green), anti-EpCAM (orange), anti-leukocyte panel (magenta) and nucleus (blue). Scale bars represent 20μm.

After establishing the dPCR analysis for isolated CTCs, we then used another aliquot of the same frozen pancreatic patients DLA to test the mutational analysis in patients CTCs. In this second round of analysis using only half of the input material, we detected CTCs in only 10% of the samples (2/19; M1 (22% (2/9)), M0 (0%, 0/10)). However, in the two samples of metastatic patients that gave the highest number of CTCs during the first test run, the presence of CTCs could be confirmed in similar numbers. In mutational analysis, both patients were *KRAS* negative while internal performance control for the mutation assays were positive (Table 2). For both patients, we sequenced the *KRAS* gene in primary tumor samples. Both tumors were wild-type for *KRAS* consistent with the CTC result (Supplementary Figure 7). In the samples of both patients we detected CTCs that were EpCAM-positive and EpCAM-negative showing the heterogeneity of CTC populations (Figure 5 and Supplementary Figure 6).

**Table 2:** Number of detected CTCs isolated from frozen DLA of pancreatic cancer patients for mutational analysis in sample set B (1 vial, 1 x 10^8^ cells/mL), overall detection rate of CTCs was 11% (22% M1, 0% M0)

Sample ID	CTCs	KRAS mutation in CTCs	KRAS mutation in tumor
5931	0	NA	NA
5804	0	NA	NA
5549	0	NA	NA
5714	0	NA	NA
5751	0	NA	NA
5902	0	NA	NA
6033	0	NA	NA
6104	0	NA	NA
5689	0	NA	NA
6017	0	NA	NA
5447	0	NA	NA
5580	0	NA	NA
5792	0	NA	NA
6012	0	NA	NA
6098	0	NA	NA
6016	0	NA	NA
5539	12	wildtype	wildtype
5803	0	NA	NA
5904	8	wildtype	wildtype

## DISCUSSION

In this study we show the superiority of filtering CTCs in comparison to EpCAM-dependent capturing of CTCs. Using spike-in samples of well characterized cultured cells, we obtained very clear results showing that filtration is superior to EpCAM-dependent enrichment especially for EpCAM low expressing cells. However, using this approach for clinical samples we still obtained a relatively low frequency of CTC positive patients. We detected CTCs in 8/19 cases in the first round of analysis. In the second analysis with less available input material, we could confirm the presence of CTCs in the two metastatic disease cases that also showed high CTC counts in the first analysis. The low frequency and number of CTCs despite the use of large amount of input material shows the limited clinical utility of this system in pancreatic cancer. Isolation of CTCs in pancreatic cancer has been recently reviewed [9]. This comprehensive overview reported a remarkable variation in detection rates and numbers of CTCs per ml between the different approaches tested. The reported detection rates range from 5% to 100% with a huge variability, essentially allowing no conclusion on the most suitable method. Interestingly, the highest detection rates were reported using EpCAM-based methods.

The isolation of CTCs from patient samples faces several challenges: 1) Pre-analytical conditions (blood sampling, handling, storage and shipment), 2) Capturing of all phenotypical CTC subtypes, 3) Unequivocal definition of true CTCs by subsequent downstream analysis.

Ad 1) Eight to ten milliliter whole blood is the commonly used source for isolation of CTCs**.** The question on how to store unprocessed patient samples for later central analysis still remains unclear. One solution is the use of frozen diagnostic leukapheresis product, the enrichment of peripheral blood mononuclear cells. Diagnostic leukapheresis was already described as detection method for CTC as the number of CTCs and cases of CTC-positive patients increased also in early stages [37]. The use of DLA product as source for CTC isolation has been described and was also validated in this work (Figure 3). However, the way of blood sampling may not be optimal for pancreatic cancer. We hypothesize that peripheral blood from the cubital vein is not the optimal source for CTC isolation especially for pancreatic cancer because the CTCs from the pancreas will be transported to the portal vein first and have to pass the liver and may be filtered out already. This is supported by the fact that the 92% CTC positive cases were observed by Gall *et al.* using portal vein blood and the CellSearch platform [38]. In line with these data, Catenacci *et al.* detected CTCs in 100% of portal vein samples (18/18) but only in 22% of the peripheral blood samples [39]. The fact that CTCs originating from the pancreas have to pass one more capillary system before entering the main circulation may be a reason for lower CTC counts in pancreatic cancer. Compared to other indications, pancreatic cancer showed the lowest CTC prevalence [13, 31]. Potentially the liver is especially effective in filtering out CTCs. This is supported by Bissolati and colleagues who found that CTC counts in portal-vein blood predicted liver metastasis [40]. In addition to blood sampling, consistent fixation protocols and preanalytics are necessary for optimal analytical performance and comparability of the results.

Ad 2) EpCAM low expressing cells are missed by EpCAM-dependent methods so that only the subpopulation of EpCAM high expressing CTCs is captured. In agreement with the earlier study of Akita *et al.* who showed that half of the tumors express EpCAM in low or non-detectable levels [18], expression of the extracellular domain of EpCAM was found in only 79% of pancreatic tumor samples [41] and a detailed analysis in a different sample set showed that 29% of the tumors were negative and 36% only weakly expressing EpCAM [42]. The loss of epithelial antigens in a large fraction of pancreatic tumors is supported by the finding that E-Cadherin expression is lost in 53% of pancreatic tumors [43]. Filtration is an alternative to enrich larger tumor cells over the mostly smaller blood cells [44]. CTCs expressing EpCAM at variable levels are isolated by a filtration based method so that all CTCs with different surface marker should be isolated. The used filter contains pores with a size of 8μm. The performance of such device is also depending on cellular rigidity and applied pressure which may explain differences between the two different prototypes used in this study. Potentially the 8μm pore size may be too large for some pancreatic CTCs although it seemed suitable during the validation with cell lines. Spike-in cell lines do not reflect the patients CTCs but help to determine the technical performance of the method [11]. Recovery of CTCs by filtration might also be hindered by apoptosis as cells shrink during this process. This has already been described in breast and prostate cancer [45]. Recently, isolation of pancreatic CTCs by both size and EpCAM-independent negative selection was described and showed very promising results [46].

Another filtration based system, the ISET method has been compared to the CellSearch system [31]. In that well designed study, ISET detected higher CTC counts compared to CellSearch but the CTCs detected by ISET were only characterized as CD45-negative larger cells. Although in this study, all tumors were EpCAM-positive, the CTCs showed huge variability of EpCAM and even the cytokeratin expression varied dramatically, showing the difficult situation with the strong heterogeneity of circulating tumor cells isolated from patient samples in contrast to cultured cells. Although ISET detected more CTCs than CellSearch, not even a trend for a correlation with progression-free survival or overall-survival was found. This is a strong argument against the clinical utility of the approach at least for pancreatic cancer. The lack of prognostic relevance together with the lacking consensus definition of a true CTC underlines the need for better characterization of CTCs in order to prove their tumor origin.

Ad 3) Concerning the clear identification of CTCs, it has to be pointed out that the majority of the CTC studies still rely on the visual identification of cells as stained or polymorph structures. Since staining intensity is highly dependent on the antigen expression and the quality of the used antibody there is no clear reference. Here, we tried to overcome the variety on staining by the usage of an antibody panel to increase staining and therefore facilitate the enumeration of CTCs. Although these improved methods showed benefits in studies with spiked cells, the number of CTC-positive patient samples remained low. EpCAM-independent methods should cope with CTCs originating of EpCAM-negative tumors or the loss of epithelial characteristics during EMT. However, also Cytokeratin loss may occur during EMT. For example, loss of Cytokeratin expression in Her2-FISH positive CTCs was described in the literature [47]. Recently Gao *et al.*, used chromosome 8 polyploidy as marker for pancreatic CTCs and found 96% of the isolated CTCs to be Cytokeratin-negative [46].

Therefore the clear identification of circulating tumor cells defined by other (more stable) features is required. Rhim and colleagues isolated epithelial cells in 8 of 11 patients with PDAC but detected the pancreatic specific Pdx-1 expression in only 29% of the cells isolated by EpCAM-based microfluidics [48]. The identification of *KRAS* mutations in pancreatic CTCs should be a sensitive tool to prove at least their pancreatic origin although these mutations can already be detected in intra-epithelial neoplasias of the pancreas (PanIn) and do not prove the presence of cancer [49]. However, we did not find evidence for *KRAS* mutations in the two samples that were CTC-positive. This finding was confirmed by sequencing primary tumor samples which turned out to be wild-type. We conclude that the 57% prevalence of *KRAS* mutations is already too low to use it as reliable marker for CTC confirmation. The high detection rate of 88% recently published for chromosome 8 polyploid CTCs [46] hints towards use of cancer-specific biomarkers with higher prevalence for the identification of CTCs in contrast to the merely epithelial phenotype.

In conclusion, we showed the advantage of a size based method in isolation of CTCs from pancreatic cancer and the feasibility of frozen DLA for detection of CTCs with subsequent downstream analysis in patient samples. Clinical use of circulating tumor cells in pancreatic cancer still needs a lot of efforts in ways of blood sampling, standardization of the preanalytics, the isolation procedures and definition of true CTCs with clear prove of their tumor cell identity.

## MATERIALS AND METHODS

### Patients

Whole blood of healthy donors was collected in EDTA-vacutainer (BD) by Clinical Research Services Berlin GmbH, Berlin. Donors were clinically healthy without any known neoplastic or infectious disease and provided written informed consent to use their blood samples for research purposes.

#### Diagnostic leukapheresis samples

Diagnostic Leukapheresis (DLA) was exactly performed as previously described [37]. Part of the DLA product was spun at 200 x g for 10 min and the supernatant was removed. The DLA product was adjusted to a white blood cell (WBC) concentration of 1 x 10^8^/mL with 5% human serum albumin (HSA, 20%, octopharma) in RPMI-1640. Aliquots of 1mL DLA product were mixed with 1mL freezing medium (4mL HSA, 4mL RPMI and 2mL DMSO), frozen in a freezing container at -80°C over night and then transferred to storage in vaporous phase of liquid nitrogen.

The use of DLA for CTC screening of an increased blood volume was approved by the ethics committee of the Heinrich-Heine-University Hospital Duesseldorf. All participating patients and healthy donors gave written informed consent.

### Methods

#### Cell culture and spike-in of cells

Cell lines used for this study were obtained from ATCC and DSMZ. Capan1 was cultured in RPMI1640 + 20% FBS + 1% Glutamin, BxPc3 in RPMI1640 + 10% FBS + 1% Glutamin, Panc1 in DMEM + 10% FBS + 1% Glutamin and SkBr3 in McCoys 5a +10% FBS +1% Glutamin at 37°C and 5% CO_2_. Prior spiking, cells were washed once with 1x PBS (Gibco) and detached at 85% confluence by Accutase solution (Sigma). Cells were spun 5 min at 127 x g, resuspended in 1 x PBS and strained through a 35μm strainer to enrich the amount of single cells.

0, 1, 3, 10 and 30 cells were transferred manually in triplicates into 10mL whole blood of healthy donors collected in EDTA-vacutainer (BD) and processed. The same number of cells was spiked in frozen healthy DLA in the vial directly after thawing. Cells were also spiked in freshly prepared buffy coat containing the same cell count as DLA, frozen and thawed for analysis.

#### Fluorescence staining

Detached cells were counted by CASY (Innovatis) and fixed by addition of Transfix (Caltag Medsystems) at a ratio of 1:20 for 45 min. Cells were spun on slides by cytospin Universal 320 (Hettich Lab). In brief, 1 x 10^5^ cells were used per cytochamber (3 Chambers per slide) and centrifuged for 7 min at 750 x g with break on. Liquid was removed and then spun again at 100 x g for 1 minute with break off. Slides were dried over night at room temperature. Cells were permeabilized by 0.5% Triton-X-100 for 15 min, washed with 1 x PBS, blocked with 10% normal goat serum, 1% BSA, 0.05% Tween-20 and 0.5% blocking solution® (Candor) and stained for 45 min by either AE1-AE3 (1:100) or by the anti-Cytokeratin Alexa Fluor 488 panel [Supplementary Table 2, (AE1/AE3, C-11, A53B/A2, DC10, LPK5, each 1:100)] as well as by anti-EpCAM-Alexa Fluor 555 (VU1D9, 1:50). Cells were washed in 1 x PBS and nuclei were stained with HOECHST 33342 for 1 min following a wash step. Slides were dried at 60 °C for a maximum of 5 min and mounted with Prolong Diamond antifade mountant (Thermofisher) and examined by an epifluoresence microscope (Observer Z.1 Zeiss and AxioVision V 4.8.2.0 Software).

#### Isolation of cells from blood by filtration

Blood was processed according to the manufacturer’s instructions as described in [32]. In short, Transfix was added to 8-10mL whole blood (1:20) and incubated for 1h. Prefixed whole blood was transferred to a 50mL conical tube, EDTA-vacutainer was rinsed with 1 x PBS twice to a final volume of 20mL and subjected to the filtration device (Siemens Healthineers) (pore size 8μm). Diluted blood was filtrated and the filter was rinsed with 1 x PBS. Cells on filter were fixed with 4% paraformaldehyde in 0.1M phosphate buffer (Electron Microscopy Sciences), permeabilized with 0.5% Triton-X-100 (Sigma) and blocked as previously described. Cells were stained using the anti-Cytokeratin-Alexa Fluor 488-panel (Supplementary Table 2), anti-leukocyte-Alexa Fluor 647-panel [CD45 (MEM 28), CD66b (G10F5), CD3 (Sk7), CD14 (G1D3), CD68 (KP1), each 1:50], anti-EpCAM-Alexa Fluor 555 (VU1D9, 1:50) and Hoechst 33342. Stained filters were analyzed manually by Observer Z.1 (Zeiss) and AxioVision V 4.8.2.0 Software.

#### Isolation of cells from blood by IsoFlux (Fluxion Biosystems)

Blood was processed according to the recommendations by Fluxion Biosystems. Briefly, whole blood was separated by overlaying onto Ficoll (GE Healthcare). Blood and Ficoll were spun at 800 x g for 30 min with breaks off. Buffy coat and plasma were transferred to a new tube and centrifuged at 280 x g for 10 min. Supernatant was removed and the pellet was loosened and transferred to a 2mL tube. EpCAM-coated beads were added. Cells and bead mixture were incubated for 2 h with overhead rotation. The bead suspension was then loaded into the cartridge and cells were isolated automatically by the device. After isolation cells were stained as previously described.

#### Isolation of cells from frozen DLA

Frozen DLA-product was thawed at 37°C in the cryovial and transferred to a 50mL tube with 1000U of Cyanase (Ribosolution) and 1mL pre-warmed thaw-solution (1:10 CTL-Wash Supplement in RPMI-1640) added dropwise to the DLA sample. In total 15.8mL Thaw Solution were added to a final volume of 20mL as follows: First, 9mL Thaw solution were slowly added dropwise. Cell suspension was incubated for 10 min at room temperature and cells were counted by Türks-Staining (Merck). Then, 6.8mL Thaw solution and 20μL cyanase inactivating protein (Ribosolution) was added (see also Supplementary Figure 8); tubes were incubated at room temperature for 20 min. 2mM EDTA (Life Technologies) and 1:20 Transfix was added. Tubes were incubated for 1h and then directly subjected to the filtration unit. For the first round of analysis (experimental setup A, 2 x 10^8^ cells/mL), an early prototype of the Siemens Device was used. For experimental setup B (using 1 x 10^8^ cells/mL), we had to use a second-generation filtration device. The pore size of the used filters was 8μm for both devices. Cells were filtered and stained as described above and in [32].

#### Mutational analysis of isolated cells by digital PCR

Isolated cells were analyzed manually by fluorescence microscopy using a Zeiss Observer Z.1 and punched from filter with a 2mm Biopsy punch (pfm medical). The punch was controlled for presence of the CTC, transferred to a thin walled PCR tube and frozen until further use. Once shortly thawed, cells on punshes were subjected to whole genome amplification (Ampli1 WGA Kit, Silicon Biosystems), 1-fold reaction mix for whole blood samples and 5-fold reaction mix for DLA-samples. WGA-DNA solution was purified by Amicon Ultra 0.5mL centrifugal filter (Merck Millipore)**.** In brief, WGA solution was added to the filter, washed twice with TE and eluted. Pure DNA was quantified by Broad Range High sensitivity DNA Assay and Qubit (Life Technologies). 50ng DNA was used for *KRAS* G12D and G12V by digital PCR (Quantstudio 3D, Thermofisher) as described in detail [50].

#### Tumor DNA preparation

Tumor tissues were marked on standard H&E-stained histologic slides. Afterwards, unstained serial sections of tumor tissues were mounted onto glass slides and macro-dissected for DNA extraction. Every macro-dissected tumor sample was cross-checked confirming that the percentage of tumor tissue was at least 80%. The extracted tumor cells were dissolved in a total volume of 190 μL digestion buffer (DNA tissue mini kit, Qiagen) and were treated with proteinase K overnight at 56°C. DNA purification was achieved using a nucleic acid robot device (BIO 101, Qiagen).

#### Sequence analysis

For sample 5539, PCR amplification was done in a total volume of 20μL containing 20ng genomic DNA, 0.2 mmol/L deoxynucleotide triphosphates, 0.5 units of Taq polymerase (HotStar Taq, QIAGEN), and the following k-ras primers: Fwd_5′-AGGCCTGCTGAAAATGACTGAA-3′, Rev_5′-AAAGAATGGTCCTGCACCAG-3′. Cycle sequencing analysis of PCR fragments was done with the BigDye Terminator system (PE Biosystems) using amplification primers for bidirectional sequencing. The reaction products were analyzed on an ABI PRISM 3700 sequencer (PE Biosystems).

For sample 5903, sequencing was done at GATC Biotech (Konstanz, Germany) using DreamTaqGreen Master Mix (ThermoFisher). Primers used: 5`CCTTATGTGTGACATGTTCTAATATAGT, 3` TTGGATCATATTCGTCCACAA. (167 bp PCR product, Tm 57.5°C). PCR conditions: 0.2μM Primers each, 0.5μL DNA Template. Cycling conditions: 95°C for 3 min; 95°C for 30 sec, 57.5°C for 30 sec, 72°C for 20 sec, repeat 30 times and final elongation at 72°C for 5 min.

## SUPPLEMENTARY MATERIALS FIGURES AND TABLES



## Abbreviations

CTC: circulating tumor cells
DLA: diagnostic leukapheresis
EpCAM: epithelial cell adhesion molecule
PanIn: pancreatic intraepithelial neoplasia
PDAC: pancreatic ductal adenocarcinoma
PFS: progression-free survival
OS: overall survival
EMT: epithelial mesenchymal transition
*KRAS*: Kirsten rat sarcoma viral oncogene homolog

## References

[1] Rahib L, Smith BD, Aizenberg R, Rosenzweig AB, Fleshman JM, Matrisian LM (2014). Projecting cancer incidence and deaths to 2030: the unexpected burden of thyroid, liver, and pancreas cancers in the United States. Cancer Res.

[2] Siegel RL, Miller KD, Jemal A (2016). Cancer statistics, 2016. CA Cancer J Clin.

[3] Yachida S, Jones S, Bozic I, Antal T, Leary R, Fu B, Kamiyama M, Hruban RH, Eshleman JR, Nowak MA, Velculescu VE, Kinzler KW, Vogelstein B, Iacobuzio-Donahue CA (2010). Distant metastasis occurs late during the genetic evolution of pancreatic cancer. Nature.

[4] Agarwal B, Correa AM, Ho L (2008). Survival in pancreatic carcinoma based on tumor size. Pancreas.

[5] Sakorafas GH, Sarr MG (2003). Pancreatic cancer after surgery for chronic pancreatitis. Dig Liver Dis.

[6] Klapman J, Malafa MP (2008). Early detection of pancreatic cancer: why, who, and how to screen. Cancer Control.

[7] Goonetilleke KS, Siriwardena AK (2007). Systematic review of carbohydrate antigen (CA 19-9) as a biochemical marker in the diagnosis of pancreatic cancer. Eur J Surg Oncol.

[8] Riva F, Dronov OI, Khomenko DI, Huguet F, Louvet C, Mariani P, Stern MH, Lantz O, Proudhon C, Pierga JY, Bidard FC (2016). Clinical applications of circulating tumor DNA and circulating tumor cells in pancreatic cancer. Mol Oncol.

[9] Nagrath S, Jack RM, Sahai V, Simeone DM (2016). Opportunities and challenges for pancreatic circulating tumor cells. Gastroenterology.

[10] Alix-Panabieres C, Pantel K (2016). Clinical applications of circulating tumor cells and circulating tumor DNA as liquid biopsy. Cancer Discov.

[11] Stoecklein NH, Fischer JC, Niederacher D, Terstappen LW (2016). Challenges for CTC-based liquid biopsies: low CTC frequency and diagnostic leukapheresis as a potential solution. Expert Rev Mol Diagn.

[12] Alix-Panabieres C, Pantel K (2014). Technologies for detection of circulating tumor cells: facts and vision. Lab Chip.

[13] Allard WJ, Matera J, Miller MC, Repollet M, Connelly MC, Rao C, Tibbe AG, Uhr JW, Terstappen LW (2004). Tumor cells circulate in the peripheral blood of all major carcinomas but not in healthy subjects or patients with nonmalignant diseases. Clin Cancer Res.

[14] Cristofanilli M, Budd GT, Ellis MJ, Stopeck A, Matera J, Miller MC, Reuben JM, Doyle GV, Allard WJ, Terstappen LW, Hayes DF (2004). Circulating tumor cells, disease progression, and survival in metastatic breast cancer. N Engl J Med.

[15] Danila DC, Heller G, Gignac GA, Gonzalez-Espinoza R, Anand A, Tanaka E, Lilja H, Schwartz L, Larson S, Fleisher M, Scher HI (2007). Circulating tumor cell number and prognosis in progressive castration-resistant prostate cancer. Clin Cancer Res.

[16] Cohen SJ, Punt CJ, Iannotti N, Saidman BH, Sabbath KD, Gabrail NY, Picus J, Morse M, Mitchell E, Miller MC, Doyle GV, Tissing H, Terstappen LW, Meropol NJ (2008). Relationship of circulating tumor cells to tumor response, progression-free survival, and overall survival in patients with metastatic colorectal cancer. JClin Oncol.

[17] Went PT, Lugli A, Meier S, Bundi M, Mirlacher M, Sauter G, Dirnhofer S (2004). Frequent EpCam protein expression in human carcinomas. Human Pathol.

[18] Akita H, Nagano H, Takeda Y, Eguchi H, Wada H, Kobayashi S, Marubashi S, Tanemura M, Takahashi H, Ohigashi H, Tomita Y, Ishikawa O, Mori M, Doki Y (2011). Ep-CAM is a significant prognostic factor in pancreatic cancer patients by suppressing cell activity. Oncogene.

[19] Fong D, Steurer M, Obrist P, Barbieri V, Margreiter R, Amberger A, Laimer K, Gastl G, Tzankov A, Spizzo G (2008). Ep-CAM expression in pancreatic and ampullary carcinomas: frequency and prognostic relevance. J Clin Pathol.

[20] Earl J, Garcia-Nieto S, Martinez-Avila JC, Montans J, Sanjuanbenito A, Rodriguez-Garrote M, Lisa E, Mendia E, Lobo E, Malats N, Carrato A, Guillen-Ponce C (2015). Circulating tumor cells (Ctc) and kras mutant circulating free Dna (cfdna) detection in peripheral blood as biomarkers in patients diagnosed with exocrine pancreatic cancer. BMC Cancer.

[21] Court CM, Ankeny JS, Hou S, Tseng HR, Tomlinson JS (2015). Improving pancreatic cancer diagnosis using circulating tumor cells: prospects for staging and single-cell analysis. Expert Rev Mol Diagn.

[22] Ankeny JS, Court CM, Hou S, Li Q, Song M, Wu D, Chen JF, Lee T, Lin M, Sho S, Rochefort MM, Girgis MD, Yao J (2016). Circulating tumour cells as a biomarker for diagnosis and staging in pancreatic cancer. Br J Cancer.

[23] Bidard FC, Huguet F, Louvet C, Mineur L, Bouche O, Chibaudel B, Artru P, Desseigne F, Bachet JB, Mathiot C, Pierga JY, Hammel P (2013). Circulating tumor cells in locally advanced pancreatic adenocarcinoma: the ancillary CirCe 07 study to the LAP 07 trial. Ann Oncol.

[24] Khan MS, Kirkwood A, Tsigani T, Garcia-Hernandez J, Hartley JA, Caplin ME, Meyer T (2013). Circulating tumor cells as prognostic markers in neuroendocrine tumors. J Clin Oncol.

[25] Yu M, Bardia A, Wittner BS, Stott SL, Smas ME, Ting DT, Isakoff SJ, Ciciliano JC, Wells MN, Shah AM, Concannon KF, Donaldson MC, Sequist LV (2013). Circulating breast tumor cells exhibit dynamic changes in epithelial and mesenchymal composition. Science.

[26] Gorges TM, Tinhofer I, Drosch M, Rose L, Zollner TM, Krahn T, von Ahsen O (2012). Circulating tumour cells escape from EpCAM-based detection due to epithelial-to-mesenchymal transition. BMC Cancer.

[27] Kallergi G, Papadaki MA, Politaki E, Mavroudis D, Georgoulias V, Agelaki S (2011). Epithelial to mesenchymal transition markers expressed in circulating tumour cells of early and metastatic breast cancer patients. Breast Cancer Res.

[28] Rhim AD, Mirek ET, Aiello NM, Maitra A, Bailey JM, McCallister F, Reichert M, Beatty GL, Rustgi AK, Vonderheide RH, Leach SD, Stanger BZ (2012). EMT and dissemination precede pancreatic tumor formation. Cell.

[29] Khan MS, Tsigani T, Rashid M, Rabouhans JS, Yu D, Luong TV, Caplin M, Meyer T (2011). Circulating tumor cells and EpCAM expression in neuroendocrine tumors. Clin Cancer Res.

[30] Gires O, Stoecklein NH (2014). Dynamic EpCAM expression on circulating and disseminating tumor cells: causes and consequences. Cell Mol Life Sci.

[31] Khoja L, Backen A, Sloane R, Menasce L, Ryder D, Krebs M, Board R, Clack G, Hughes A, Blackhall F, Valle JW, Dive C (2012). A pilot study to explore circulating tumour cells in pancreatic cancer as a novel biomarker. Br J Cancer.

[32] Magbanua MJ, Pugia M, Lee JS, Jabon M, Wang V, Gubens M, Marfurt K, Pence J, Sidhu H, Uzgiris A, Rugo HS, Park JW (2015). A novel strategy for detection and enumeration of circulating rare cell populations in metastatic cancer patients using automated microfluidic filtration and multiplex immunoassay. PLoS One.

[33] http://cancer.sanger.ac.uk/cosmic. 2016`, January 28th #241

[34] Kulemann B, Liss AS, Warshaw AL, Seifert S, Bronsert P, Glatz T, Pitman MB, Hoeppner J (2016). KRAS mutations in pancreatic circulating tumor cells: a pilot study. Tumour Biol.

[35] Harb W, Fan A, Tran T, Danila DC, Keys D, Schwartz M, Ionescu-Zanetti C (2013). Mutational analysis of circulating tumor cells using a novel microfluidic collection device and qPCR assay. Transl Oncol.

[36] Posel C, Moller K, Frohlich W, Schulz I, Boltze J, Wagner DC (2012). Density gradient centrifugation compromises bone marrow mononuclear cell yield. PLoS One.

[37] Fischer JC, Niederacher D, Topp SA, Honisch E, Schumacher S, Schmitz N, Zacarias Föhrding L, Vay C, Hoffmann I, Kasprowicz NS, Hepp PG, Mohrmann S, Nitz U (2013). Diagnostic leukapheresis enables reliable detection of circulating tumor cells of nonmetastatic cancer patients. Proc Natil Acad Sci.

[38] Gall TM, Jacob J, Frampton AE, Krell J, Kyriakides C, Castellano L, Stebbing J, Jiao LR (2014). Reduced dissemination of circulating tumor cells with no-touch isolation surgical technique in patients with pancreatic cancer. JAMA Surg.

[39] Catenacci DV, Chapman CG, Xu P, Koons A, Konda VJ, Siddiqui UD, Waxman I (2015). Acquisition of portal venous circulating tumor cells from patients with pancreaticobiliary cancers by endoscopic ultrasound. Gastroenterology.

[40] Bissolati M, Sandri MT, Burtulo G, Zorzino L, Balzano G, Braga M (2015). Portal vein-circulating tumor cells predict liver metastases in patients with resectable pancreatic cancer. Tumour Biol.

[41] Fong D, Moser P, Kasal A, Seeber A, Gastl G, Martowicz A, Wurm M, Mian C, Obrist P, Mazzoleni G, Spizzo G (2014). Loss of membranous expression of the intracellular domain of EpCAM is a frequent event and predicts poor survival in patients with pancreatic cancer. Histopathology.

[42] Fong D, Seeber A, Terracciano L, Kasal A, Mazzoleni G, Lehne F, Gastl G, Spizzo G (2014). Expression of EpCAM(MF) and EpCAM(MT) variants in human carcinomas. J Clin Pathol.

[43] Pignatelli M, Ansari TW, Gunter P, Liu D, Hirano S, Takeichi M, Kloppel G, Lemoine NR (1994). Loss of membranous E-cadherin expression in pancreatic cancer: correlation with lymph node metastasis, high grade, and advanced stage. J Pathol.

[44] Vona G, Sabile A, Louha M, Sitruk V, Romana S, Schutze K, Capron F, Franco D, Pazzagli M, Vekemans M, Lacour B, Brechot C, Paterlini-Brechot P (2000). Isolation by size of epithelial tumor cells: a new method for the immunomorphological and molecular characterization of circulatingtumor cells. Am JPathol.

[45] Adams DL, Stefansson S, Haudenschild C, Martin SS, Charpentier M, Chumsri S, Cristofanilli M, Tang CM, Alpaugh RK (2015). Cytometric characterization of circulating tumor cells captured by microfiltration and their correlation to the CellSearch((R)) CTC test. Cytometry A.

[46] Gao Y, Zhu Y, Zhang Z, Zhang C, Huang X, Yuan Z (2016). Clinical significance of pancreatic circulating tumor cells using combined negative enrichment and immunostaining-fluorescence *in situ* hybridization. J Exp Clin Cancer Res.

[47] Pecot CV, Bischoff FZ, Mayer JA, Wong KL, Pham T, Bottsford-Miller J, Stone RL, Lin YG, Jaladurgam P, Roh JW, Goodman BW, Merritt WM, Pircher TJ (2011). A novel platform for detection of CK+ and CK- CTCs. Cancer Discov.

[48] Rhim AD, Thege FI, Santana SM, Lannin TB, Saha TN, Tsai S, Maggs LR, Kochman ML, Ginsberg GG, Lieb JG, Chandrasekhara V, Drebin JA, Ahmad N (2014). Detection of circulating pancreas epithelial cells in patients with pancreatic cystic lesions. Gastroenterology.

[49] Feldmann G, Beaty R, Hruban RH, Maitra A (2007). Molecular genetics of pancreatic intraepithelial neoplasia. J Hepatobiliary Pancreat Surg.

[50] Brychta N, Krahn T, von Ahsen O (2016). Detection of KRAS mutations in circulating tumor DNA by digital PCR in early stages of pancreatic cancer. Clin Chem.

