# Supplementary material for: Isolation of circulating tumor cells from pancreatic cancer by automated filtration
Source: Oncotarget. 2017 Sep 16;8(49):86143–56. doi: 10.18632/oncotarget.21026 (PMC5689673; doi:10.18632/oncotarget.21026)
Supplement: Supplementary file 1 [file oncotarget-08-86143-s001.pdf]

Isolation of circulating tumor cells from pancreatic cancer by automated filtration

SUPPLEMENTARY MATERIALS

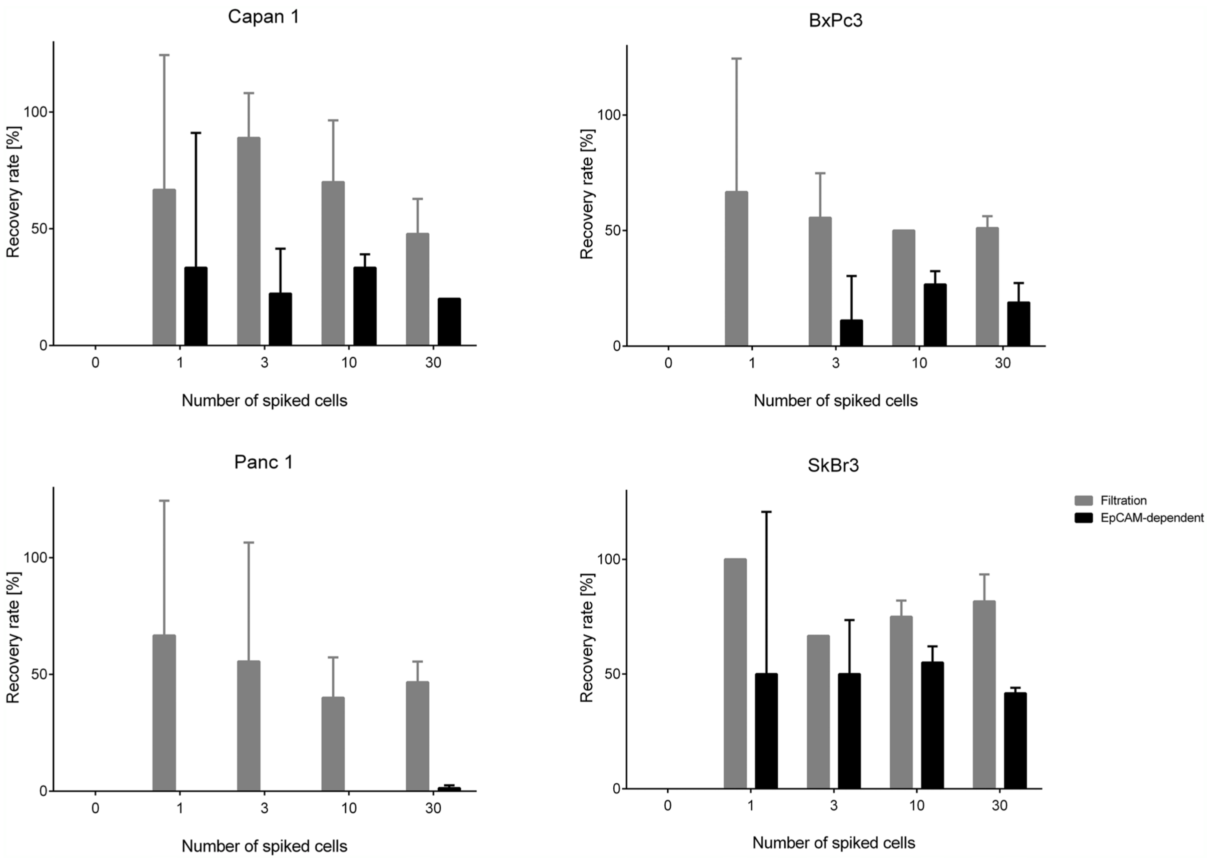

Supplementary Figure 1: Recovery rates of spike-in experiments with different cell lines in whole blood. 1, 3, 10 and 30 Capan 1 (EpCAM high), BxPc3 (EpCAM medium) and Panc 1 (EpCAM low) as well as the breast cancer cell line SkBr3 (EpCAM high) were spiked into whole blood and isolated by filtration (grey) and EpCAM dependent capture (black) (n=3 for every number of cells).

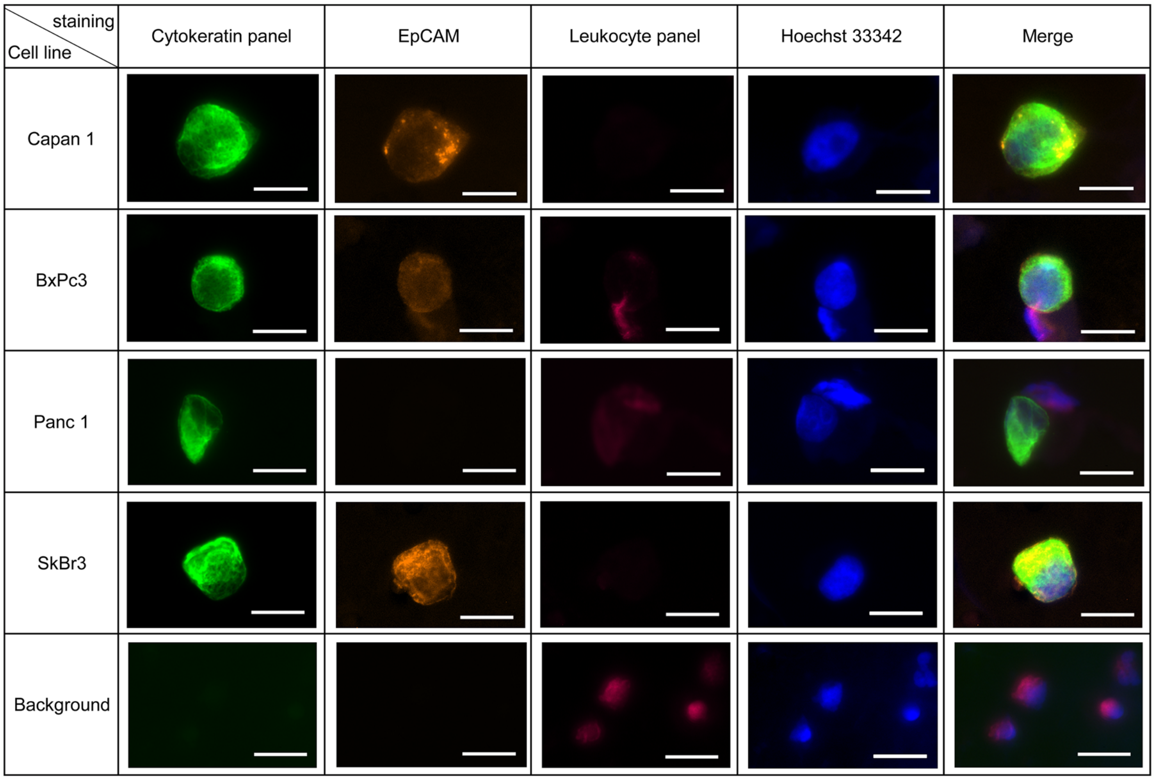

Supplementary Figure 2: Representative pictures of isolated cells from whole blood by filtration. Scale bar represent 20μm.

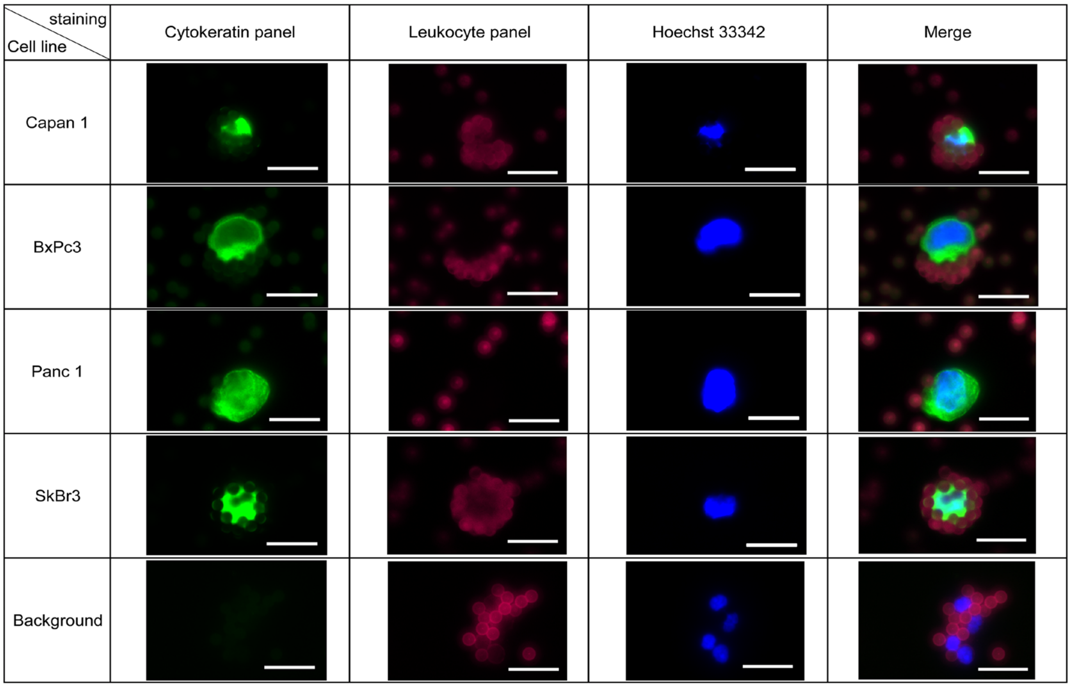

Supplementary Figure 3: Representative pictures of isolated cells from whole blood by IsoFlux. Scale bar represents 20µm.

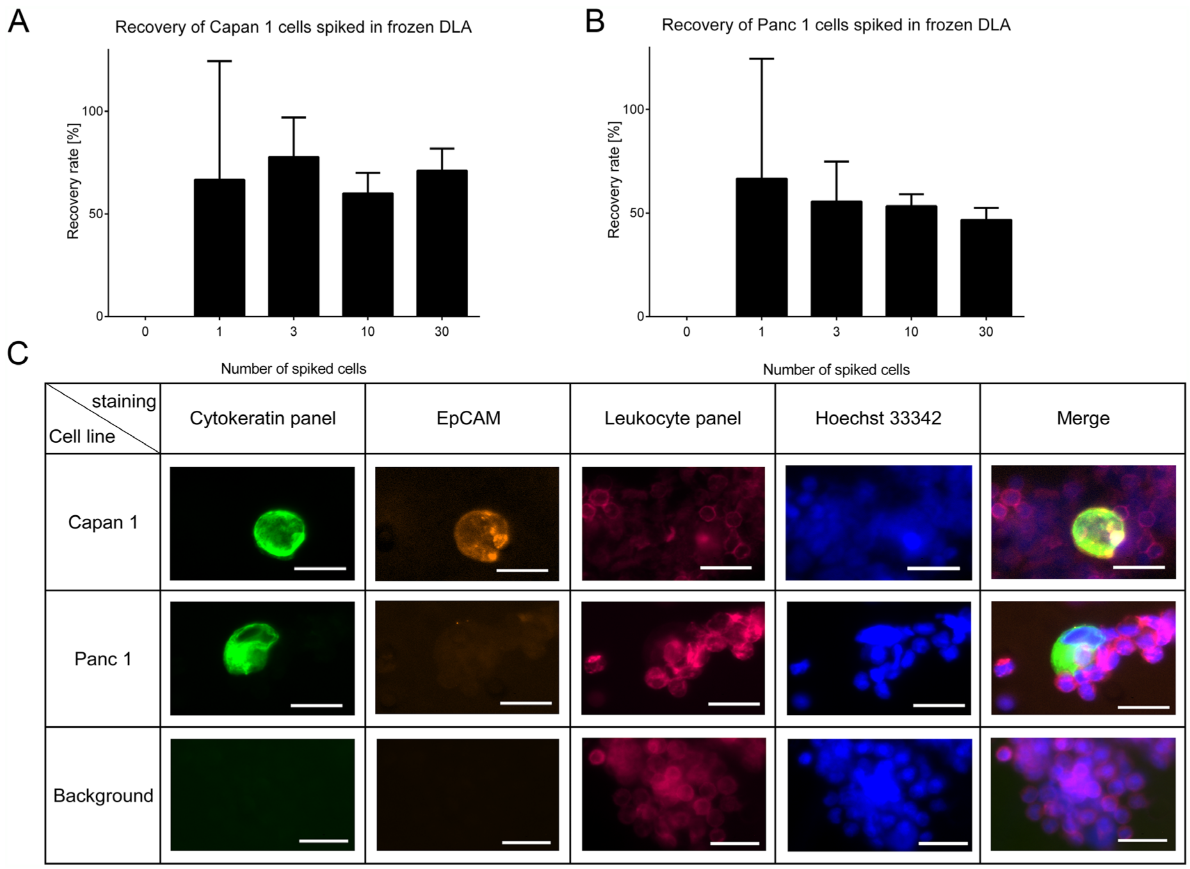

Supplementary Figure 4: (A) Recovery of Capan 1 cells spiked into frozen DLA (n=3). **(B)** Recovery rate of Panc 1 from frozen DLA (n=3). **(C)** Representative pictures of isolated cells.

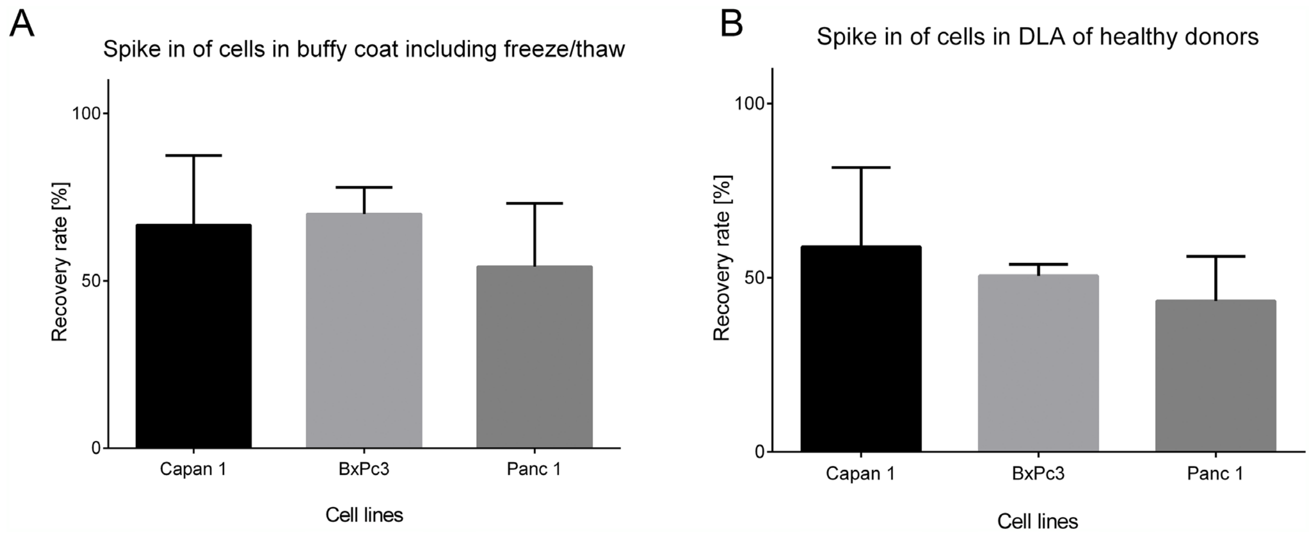

Supplementary Figure 5: No difference in recovery rate if samples were frozen. **(A)** Cells were spiked into freshly prepared buffy coat, frozen and thawn according to protocol (n=3). **(B)** Cells were spiked into thawn DLA from healthy donor (n=6).

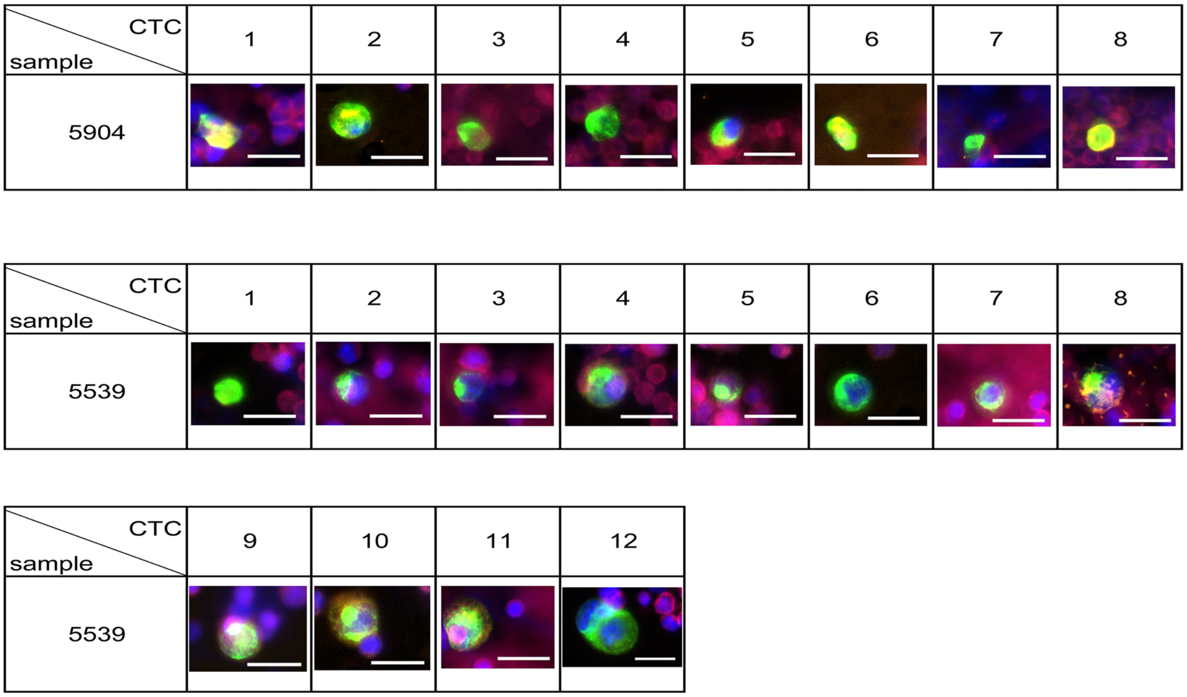

Supplementary Figure 6: Representative pictures of CTCs isolated from pancreatic cancer patients.

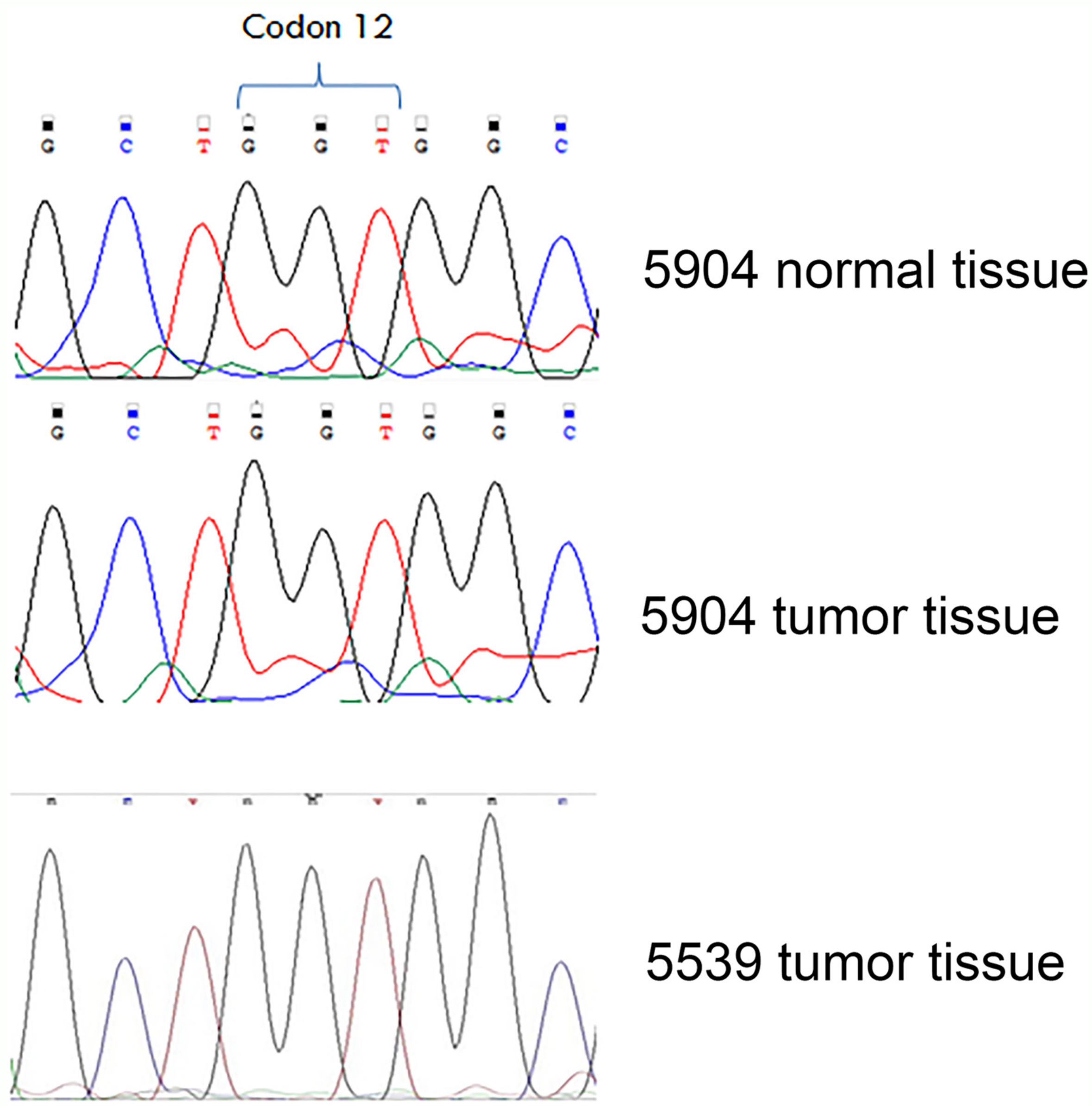

Supplementary Figure 7: KRAS codon12 sequencing data for primary tumor samples.

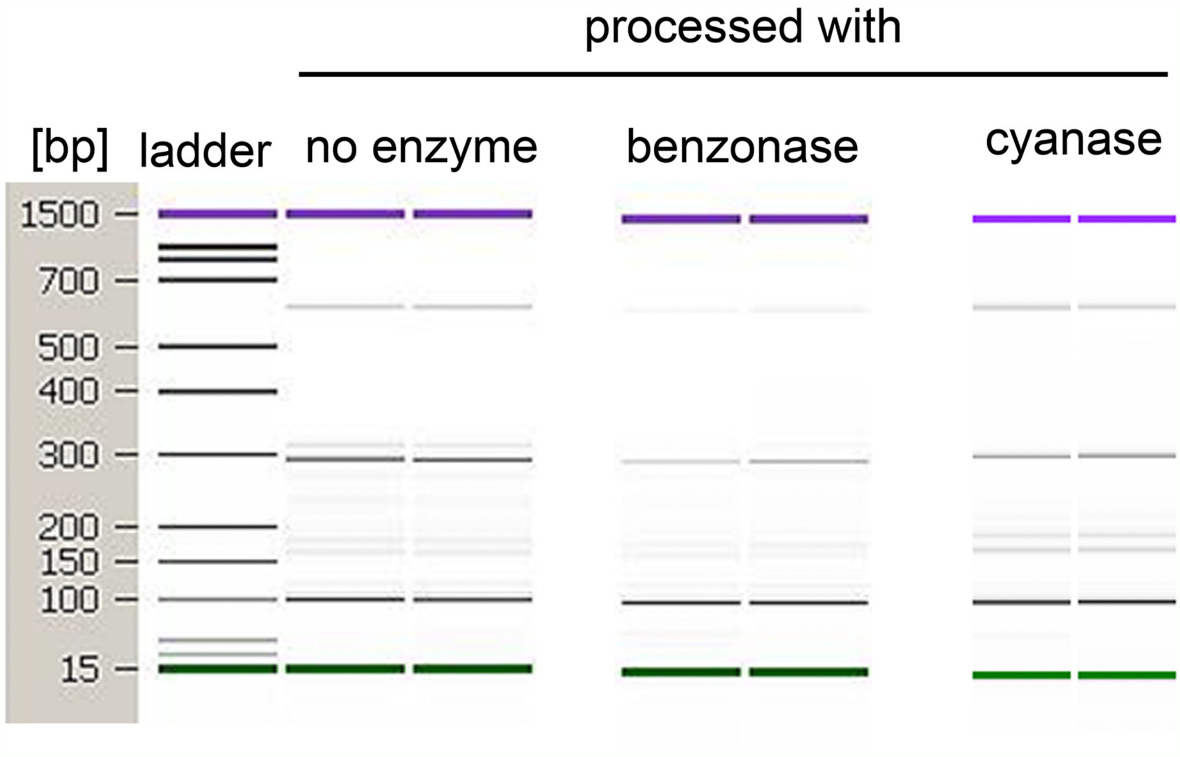

Supplementary Figure 8: DNA-quality after thawing of samples using no enzyme, Benzonase (25U/mL) and Cyanase (50U/mL). Samples were processed according to protocol on the filtration device by using different or no enzyme. Isolated cells were punshed from filter and DNA was amplified by DNA. 1µL of WGA-DNA was used for quality control (QC). QC was conducted by amplifying four genomic loci of different length (91, 108-166, 299, 614bp). As benzonase activity is not inhibited during processing, DNA is digested as seen by faint bands especially at 614 bp. This is not seen in samples using cyanase as this enzyme can be inhibited during processing. Processing with no enzyme resulted in little recovery, benzonase and cynase in similar recovery (data not shown).

Supplementary Table 1: Cytokeratin expression in pancreatic cancer. (according to Lee et al., Mod. Pathol. 16: 403-410)

Cytokeratin Cancer type	1	2	3	4	5	6	7	8	9	10	11	12	13	14	15	16	17	18	19	20
Pancreatic cancer							x	x					x					x	x	

Supplementary Table 2: Cytokeratin antibody panel

Cytokeratin antibody	1	2	3	4	5	6	7	8	9	10	11	12	13	14	15	16	17	18	19	20
AE1-AE3	x	x	x	x	x	x	x	x		x				x	x	x			x	
C-11				x	x	x		x		x			x					x		
A53-B/A2																			x	
DC10																		x		
LP5K							x													
